# A deep learning-based prognostic model for diffuse large B-cell lymphoma incorporating PET/CT imaging features

**DOI:** 10.3389/fonc.2026.1849942

**Published:** 2026-06-16

**Authors:** Man Wang, Siyuan Wu, Qishan Cen, Haiyan Yang, Shengsheng Zhou, Jinfeng Qiu, Shengcai Huang, Zhigang Peng

**Affiliations:** 1 Department of Oncology, The First Affiliated Hospital of Guangxi Medical University, Nanning, China; 2 Education Department of Guangxi Zhuang Autonomous Region, Key Laboratory of Hematology, Guangxi Medical University, Nanning, China; 3 Guangxi Medical University, Nanning, China; 4 Department of Nuclear Medicine, The First Affiliated Hospital of Guangxi Medical University, Nanning, China

**Keywords:** deep features, diffuse large B-cell lymphoma, fusion model, machine learning, prognostic prediction

## Abstract

**Objective:**

This study aims to create and validate a prognostic prediction model that leverages deep features derived from PET/CT imaging to support personalized precision treatment for patients with diffuse large B-cell lymphoma (DLBCL).

**Materials and methods:**

We retrospectively analyzed clinical and pretreatment PET/CT data from 209 patients with DLBCL. Deep features were extracted from three-dimensional tumor lesions; after dimensionality reduction, a radiomics model was built to predict 3-year overall survival (OS). We evaluated six machine learning algorithms: Logistic Regression (LR), Support Vector Machine (SVM), K-Nearest Neighbors (KNN), Random Forest (RF), eXtreme Gradient Boosting (XGBoost), and Light Gradient Boosting Machine (LightGBM). The optimal radiomics model was integrated with clinical features to form a fusion model. The fusion model’s performance was assessed on both training and independent test sets using metrics such as accuracy, area under the receiver operating characteristic (ROC) curve (AUC), sensitivity, and specificity. Furthermore, the model’s clinical utility was evaluated through Decision Curve Analysis (DCA), and survival analysis was performed using the Kaplan-Meier (KM) method.

**Results:**

Univariate and multivariate analyses identified age, AB group, International Prognostic Index (IPI) score, serum β2-microglobulin level, and maximum tumor diameter as independent risk factors for 3-year survival in DLBCL patients. Among the machine learning-based radiomics models, the LR model showed superior predictive performance, achieving an accuracy of 0.865, AUC of 0.950, sensitivity of 0.875, and specificity of 0.863. Integration with clinical features further improved the model’s performance. On the test set, the fusion model attained an accuracy of 0.921, an impressive AUC of 0.974, and sensitivities and specificities of 0.846 and 0.940, respectively. DCA revealed that this fusion model offers a significant clinical net benefit for prognostic risk prediction in DLBCL patients over a wide threshold probability range of 0.05–0.900.

**Conclusion:**

The fusion model, combining PET/CT deep features with clinical characteristics, is a dependable prognostic tool for DLBCL, holding substantial promise for clinical application and personalized treatment.

## 1 Introduction

Diffuse large B-cell lymphoma (DLBCL) is the most common aggressive subtype of non-Hodgkin lymphoma (NHL), accounting for approximately 30%-40% of NHL cases, and is characterized by significant heterogeneity in clinical outcomes (1, 2). Although immunochemotherapy, particularly the R-CHOP regimen, has substantially improved overall survival (OS), approximately 30%-40% of patients still experience primary resistance or relapse, with a 5-year OS rate of only approximately 30% in high-risk patients (3, 4). In the rituximab era, traditional prognostic systems for DLBCL have proven inadequate for meeting the demands of clinical prognosis prediction. The International Prognostic Index (IPI) has limited discriminatory power for identifying high-risk patients and fails to fully capture molecular heterogeneity and disease complexity (5). Therefore, there is an urgent need to develop a more comprehensive and accurate prognostic assessment system to optimize individualized treatment strategies for patients with DLBCL.

As a crucial imaging assessment tool, PET/CT is not only used for lymphoma staging and response evaluation, but its metabolic parameters, such as baseline metabolic tumor volume (MTV), total lesion glycolysis (TLG), and maximum standardized uptake value (SUVmax), also serve as independent prognostic predictors (6, 7). However, owing to the highly heterogeneous biological behavior of DLBCL, these metabolic parameters cannot fully capture the subtle structural differences within the tumor (8). Tumor heterogeneity is a key factor contributing to drug resistance and relapse in patients with DLBCL. Consequently, establishing novel PET/CT-based radiomic prognostic biomarkers that accurately reflect complex intratumoral heterogeneity is crucial for accurate prognostication in DLBCL. With ongoing advances in medical imaging technology, radiomics is increasingly playing an important role in oncology research (9–11). Traditional radiomic features (e.g., texture and shape) are largely manually designed, suffer from feature redundancy, and have limited interpretability. In recent years, the development of artificial intelligence (AI), particularly deep learning technologies, has achieved breakthrough progress in medical image analysis, providing new tools and methods for disease diagnosis, treatment, and prognosis (12–14). Specifically, deep learning technologies can automatically extract deep spatial and textural features from medical images that are imperceptible to the human eye, thereby reducing the need for manual intervention and improving the efficiency and accuracy of feature extraction. These extracted features may capture biological information on the tumor microenvironment, heterogeneity, and treatment sensitivity (15). Studies have demonstrated that deep learning technologies hold significant promise for clinical applications in the prognostic assessment and risk stratification of various malignant tumors (16–19). In the field of DLBCL, Yang et al. developed and externally validated a combined prognostic model integrating PET radiomics, metabolic parameters, and clinical factors, improving PFS and OS prediction in elderly DLBCL patients (20). An AutoML-generated radiomics score based on ^18^F-FDG PET outperformed conventional metabolic parameters in predicting treatment response in elderly DLBCL, and a multivariable model based on it further improved predictive accuracy, offering potential clinical decision support (21).

**Figure 1:**
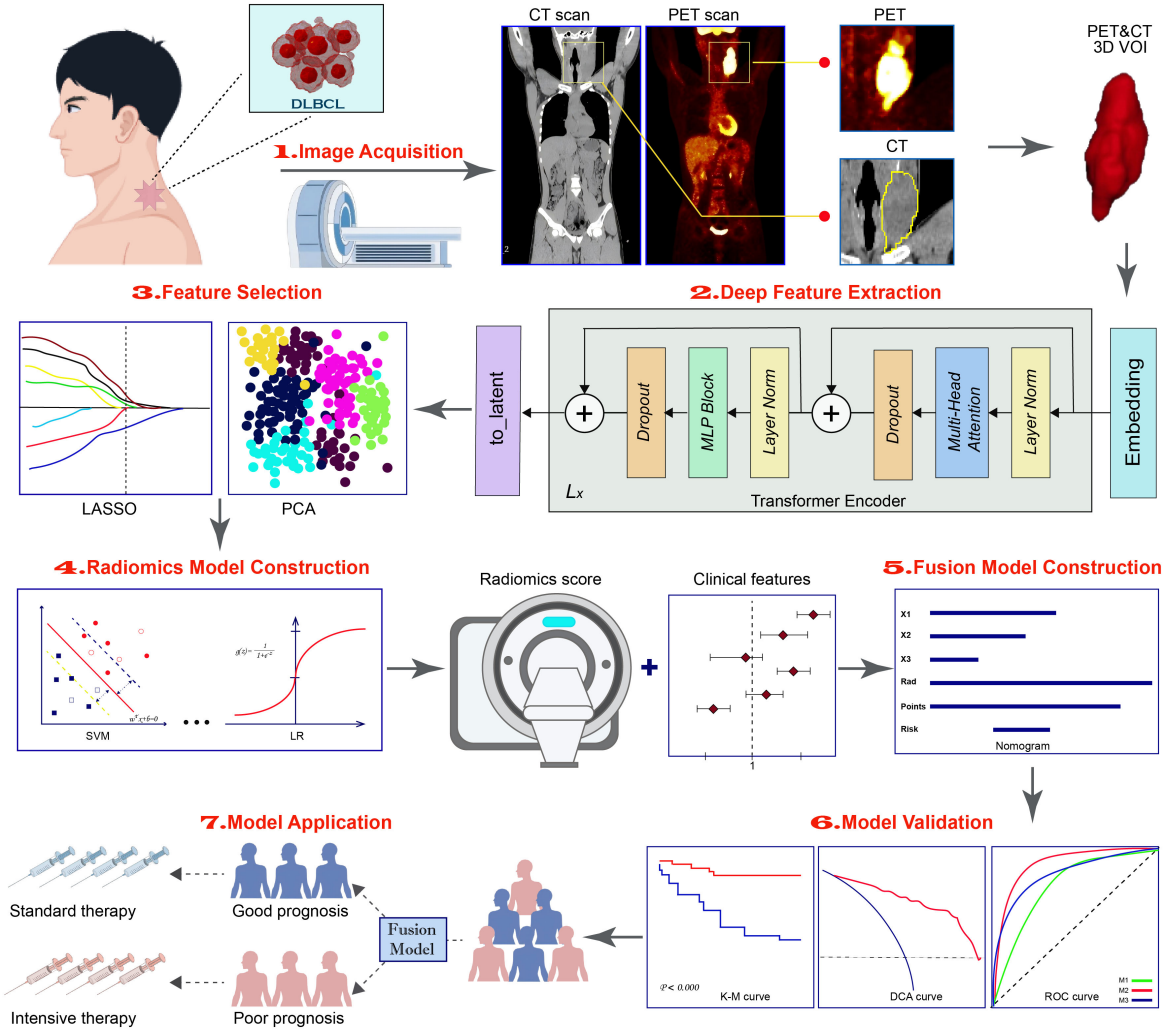
Schematic flowchart of study design.

While there is growing interest in integrating PET/CT-derived deep learning features with clinical information, the potential of such integrated models to deliver comprehensive and generalizable prognosis prediction in DLBCL remains underexplored. Therefore, this study aimed to integrate deep-learning-extracted PET/CT imaging features with routine clinical parameters to construct a DLBCL prognostic prediction model with superior performance and enhanced generalizability. The technical workflow is as follows: First, a transformer-based model framework was employed to automatically extract high-throughput deep imaging features from baseline PET/CT images. Subsequently, these deep features were fused across modalities using key clinical characteristics. Finally, machine learning algorithms were applied to select the most predictive feature combinations and construct a clinical imaging fusion model. This model is primarily intended to predict the 3-year overall survival probability for newly diagnosed DLBCL patients, providing objective quantitative evidence for early treatment intensification in high-risk patients and de-escalation strategies in low-risk patients. It aims to offer scientific guidance to clinicians in formulating individualized treatment plans for DLBCL, thereby facilitating precision intervention (the study design flowchart is shown in **Figure 1**).

## 2 Materials and methods

### 2.1 Study population and selection criteria

This study was approved by the Ethics Committee of the First Affiliated Hospital of Guangxi Medical University (Approval No. 2024-S056-01). All patient data were anonymized to ensure confidentiality. The study cohort retrospectively comprised treatment-naive patients with DLBCL who were diagnosed and treated at the Departments of Oncology and Hematology at the First Affiliated Hospital of Guangxi Medical University between January 2018 and January 2022.

The inclusion criteria were as follows: (1) age ≥ 18 years; (2) a confirmed pathological diagnosis of DLBCL according to the International Classification of Diseases for Oncology, 3rd Edition (ICD-O-3); (3) no prior anti-lymphoma therapy; (4) treatment with a standard R-CHOP regimen as first-line therapy, with all patients planned to receive complete cycles (6 cycles); (5) availability of a pre-treatment PET/CT scan; and (6) availability of complete clinical data and at least three years of follow-up information. Patients were excluded if they (1) had a history of other primary malignancies, (2) had received prior DLBCL-specific treatment, (3) had incomplete data or significant data discrepancies, or (4) discontinued therapy prematurely due to poor adherence or intolerable toxicity.

Based on these criteria, 355 patients with DLBCL were screened. Ultimately, 209 patients (102 males and 107 females) were included in the final analysis. The 209 subjects were randomly divided into training and test sets in a 7:3 ratio. All 209 patients completed all six cycles of the standard R-CHOP regimen.

### 2.2 Data collection

This study had a retrospective design. PET/CT images were retrieved from the hospital’s imaging system and uniformly converted to the DICOM format. The corresponding clinical data were collected from the electronic medical record system of the center. Overall survival (OS) was defined as the time from the date of diagnosis to death from any cause or to the last follow-up date. Patient survival status, the primary outcome measure, was obtained through medical record review (including death records) and telephone follow-up interviews. The follow-up cutoff date was set as February 1, 2025.

### 2.3 Clinical feature extraction and selection

For the clinical feature analysis, the following baseline patient characteristics were collected: gender, age, molecular subtype, disease stage (Ann Arbor stage), International Prognostic Index (IPI) score, hemoglobin level, absolute neutrophil count, absolute lymphocyte count, absolute monocyte count, lactate dehydrogenase level, serum β2-microglobulin level, maximum tumor diameter, and maximum standardized uptake value (SUVmax), among others. It should be noted that the maximum tumor diameter was defined as the longest axial diameter of the largest measurable lesion on PET/CT images. Univariate analysis was first performed to identify the potential risk factors associated with 3-year survival. Subsequently, multivariate logistic regression analysis was performed to identify independent risk factors.

### 2.4 Extraction of deep features from PET/CT images

The PET/CT images were preprocessed, including resampling, registration, and lesion segmentation. First, all scans were resampled to a consistent voxel resolution and spatially co-registered. Subsequently, two experienced PET/CT specialists manually delineated only the maximal tumor lesion (the largest PET-positive lesion per patient) by drawing three-dimensional volumes of interest using a 3D Slicer (version 5.6.2). The final contours were determined by a consensus. To assess inter-observer reproducibility, the intraclass correlation coefficient (ICC) was calculated based on 30 randomly selected cases independently segmented by both specialists before consensus. The ICC was 0.93, indicating excellent agreement.

A 3D Vision Transformer (ViT) model was employed for deep feature extraction. The model was implemented in the PyTorch deep learning framework, and cross-entropy loss was used as the optimization loss function. The model was pretrained on the ImageNet dataset, and the 2D ImageNet pre-trained weights were adaptively converted to 3D weights for direct initialization of the 3D ViT model. Each segmented lesion region was cropped to a fixed size of 64×64×48 voxels. The 3D ViT partitioned the input volume into 3D patches with a spatial patch size of 16×16 and a frame patch size of 2 and linearly embedded them into a 1024-dimensional space. The transformer encoder comprised 6 layers (depth = 6), each with 8 attention heads (embedding dimension = 1024; MLP dimension = 2048, dropout rate = 0.1). Transfer learning was performed using our own dataset, which was randomly split into training (70%) and test (30%) sets. Model training and selection were conducted with validation every 1 epoch, and the optimal model with the best validation performance was automatically selected for subsequent analysis. The model was fine-tuned on the training set for 100 epochs with a batch size of 4, using the Adam optimizer (initial learning rate = 0.001). ImageNet-based normalization was applied to the input data, the pretrained ImageNet weights were used for initialization, and no layers were frozen during training. After fine-tuning, the 1024-dimensional deep features were extracted from the final encoder layer separately for the PET and CT volumes.

To reduce the dimensionality of these high-dimensional data, we employed Principal Component Analysis (PCA) as an initial step to transform the data into a set of linearly uncorrelated components. PCA was fitted exclusively on the training set, and the same transformation was then applied to the test set. Finally, to identify the features most predictive of prognosis, the Least Absolute Shrinkage and Selection Operator (LASSO) was applied for further feature selection and regularization. LASSO was trained only on the training set, with 10-fold cross-validation used to select the optimal regularization parameter λ; the selected features were then evaluated on the test set.

### 2.5 Radiomics model construction

The selected features were used to construct machine learning models in Python (Python Software Foundation, version 3.7.2) using the Scikit-learn package. Six machine learning algorithms were employed: k-nearest neighbors (KNN), LightGBM, logistic regression (LR), random forest (RF), support vector machine (SVM), and XGBoost. All models were trained on the training set using five-fold cross-validation to determine the optimal hyperparameters and derive the final radiomics model. The best-performing model was then used to estimate each patient’s 3-year mortality probability, yielding a radiomics score.

### 2.6 Construction and validation of clinical model and fusion model

Both the clinical model and clinical-radiomic fusion model were developed using the Logistic Regression (LR) algorithm to ensure methodological consistency. The pure clinical model was established using independent clinical prognostic risk factors of DLBCL screened by multivariate regression analysis. The clinical-radiomic fusion model was constructed by integrating these independent clinical risk factors with the radiomics score derived from PET/CT images. The unified modeling strategy eliminated algorithmic bias and ensured rigorous comparability across models.

The receiver operating characteristic (ROC) curve was plotted to quantify the predictive discrimination ability of each model, and DeLong’s test was applied to compare the statistical differences in AUC values among different models. The net reclassification improvement (NRI) and integrated discrimination improvement (IDI) were calculated to quantitatively assess the incremental risk stratification and discriminative performance of the models. Calibration curves were used to verify the consistency between model-predicted probabilities and actual clinical outcomes. Decision curve analysis (DCA) was finally conducted to evaluate the clinical net benefit and practical application value of each model.

### 2.7 Statistical analysis

All statistical analyses, model construction, and validation were performed using Python. According to the results of normality tests, continuous variables were presented as mean ± standard deviation or median (interquartile range), and inter-group differences were assessed using the independent-samples t-test or the Mann-Whitney U test. Categorical variables were described as counts and percentages, and compared using the Chi-square test or Fisher’s exact test. Univariate and multivariate analyses were utilized to identify independent clinical prognostic factors for DLBCL. Kaplan-Meier survival analysis and the log-rank test were used to compare survival between high- and low-score patient subgroups. All statistical tests were two-tailed, and a *P*-value<0.05 was considered statistically significant.

## 3 Results

### 3.1 Identification of independent prognostic factors

A total of 209 patients with DLBCL were enrolled in this study. Based on their 3-year survival status, they were divided into a survivor group (n = 166) and a non-survivor group (n = 43) for the comparative analysis of baseline characteristics (**Supplementary Table 1**). Univariate analysis identified age, AB group, IPI score, serum β2-microglobulin level, and maximum tumor diameter as factors significantly associated with 3-year survival (P < 0.05). No statistically significant differences were observed for gender, GCB Subtype, Ann Arbor stage, hemoglobin(HGB), neutrophil (NEU), lymphocyte (LYM), monocyte(MON), lactate dehydrogenase (LDH), or SUVmax (*P* > 0.05).

Multivariate logistic regression analysis confirmed that age (OR = 1.424, 95% CI: 1.084–1.871), AB group (OR = 1.149, 95% CI: 0.988–1.498), IPI score (OR = 1.173, 95% CI: 0.986–1.586), serum β2-microglobulin level (OR = 1.372, 95% CI: 1.005–1.883), and maximum tumor diameter (OR = 1.339, 95% CI: 1.025–1.749) were independent risk factors for 3-year survival in DLBCL patients (*P* < 0.05). Among these, age emerged as the most significant risk factor, with the highest odds ratio (**Table 1**).

**Table 1:** Univariate and multivariate analysis of prognostic factors of DLBCL.

Characteristic	Univariate analysis			Multivariate analysis		
	OR	95%CI	P value	OR	95%CI	P value
Gender	0.793	0.631—0.998	0.097			
Age	1.576	1.232—2.014	0.002	1.424	1.084—1.871	0.033
GCB Subtype	0.944	0.751—1.186	0.679			
Ann Arbor Stage	1.306	1.035—1.647	0.059			
AB_group	1.381	1.094—1.742	0.023	1.149	0.988—1.498	0.035
International Prognostic Index (IPI)	1.663	1.303—2.121	0.001	1.173	0.986—1.586	0.021
Hemoglobin(HGB)	0.804	0.638—1.014	0.123			
Neutrophil (NEU)	1.313	1.023—1.685	0.072			
Lymphocyte (LYM)	0.96	0.758—1.217	0.777			
Monocyte(MON)	0.97	0.769—1.225	0.830			
Lactate Dehydrogenase (LDH)	1.382	1.034—1.848	0.067			
β2-Microglobulin (β2-MG)	1.763	1.302—2.387	0.002	1.372	1.005—1.883	0.011
Maximum Tumor Diameter	1.444	1.121—1.859	0.017	1.339	1.025—1.749	0.017
SUVmax	1.169	0.929—1.473	0.265			

### 3.2 Performance of the PET/CT deep learning radiomics model

**Figure 2:**
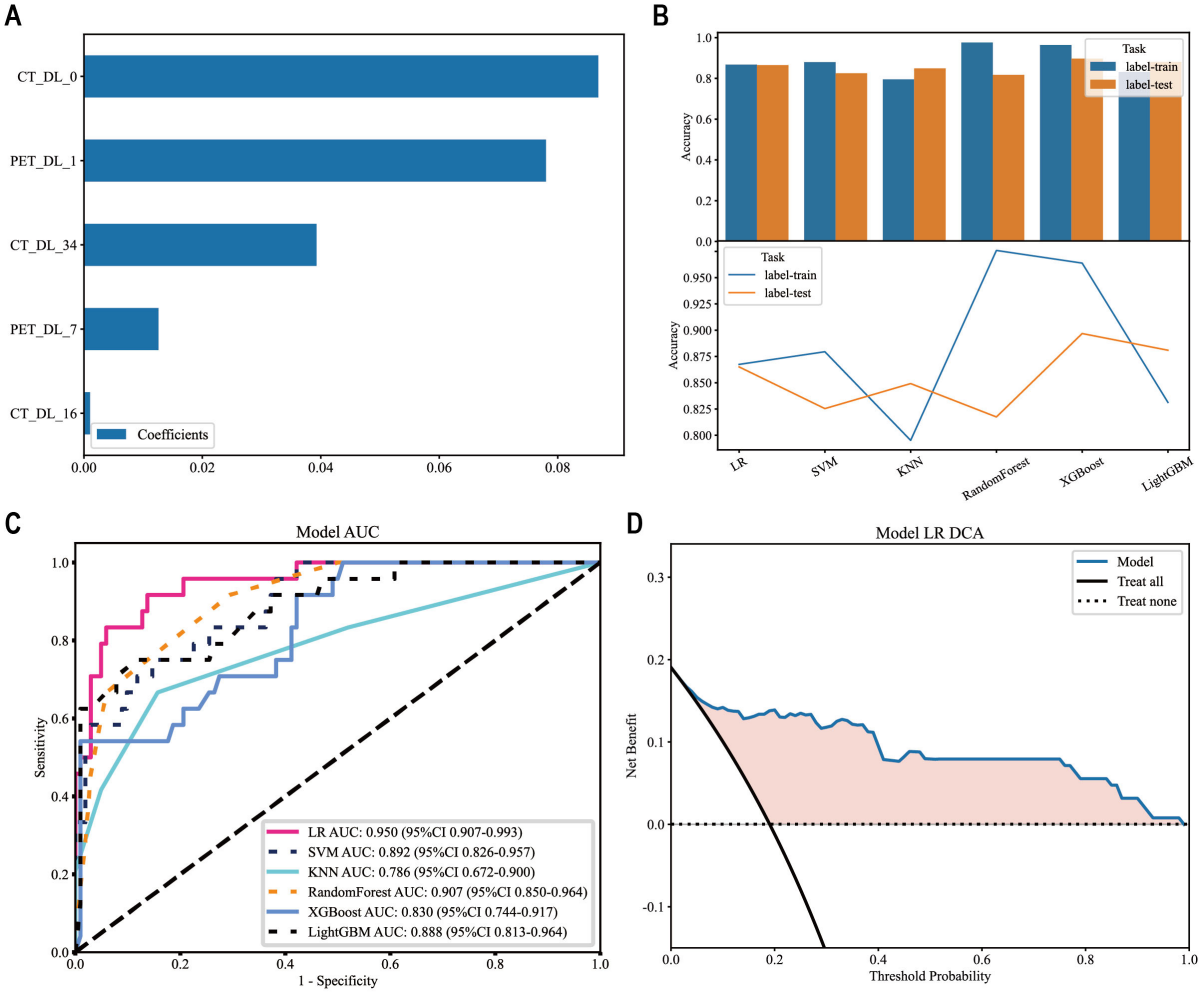
Performance comparison of machine learning models constructed based on deep features extracted from PET/CT images. **(A)** Weight distribution of the deep features selected by the LASSO regression. **(B)** Accuracy comparison of the six models. **(C)** AUC comparison of models in the test set. **(D)** Decision curve analysis (DCA) of the LR model.

The 209 eligible patients were randomly divided into a training set (n = 146) and a test set (n = 63) in a 7:3 ratio using a random-number generator. Baseline characteristics, including gender, age, disease stage, laboratory parameters, and prognosis, were well balanced between the two sets, with no statistically significant differences observed (P > 0.05; **Supplementary Table 2**). For each delineated volume of interest, 1024 deep features were initially extracted using a pretrained transformer model. Subsequently, LASSO regression was applied for dimensionality reduction and feature selection, ultimately identifying five features with the highest discriminative power: three features derived from CT images and two from PET images. The corresponding feature weights are shown in **Figure 2A**.

Six machine learning models (LR, SVM, KNN, RF, XGBoost, and LightGBM) were constructed using the selected features. On the test set, they achieved accuracies of 0.865, 0.825, 0.849, 0.817, 0.897, and 0.881, respectively. The corresponding AUC (95% CI) were 0.950 (0.907–0.993), 0.892 (0.826–0.957), 0.786 (0.672–0.901), 0.907 (0.850–0.964), 0.830 (0.744–0.917), and 0.888 (0.813–0.964). Sensitivities were 0.875, 0.708, 0.417, 0.792, 0.500, and 0.708, while specificities were 0.863, 0.853, 0.951, 0.824, 0.990, and 0.922. **Supplementary Table 3** summarizes the performance of all models on the training set, and **Table 2** presents their test set performance. These results demonstrate that the LR model exhibited superior predictive performance for DLBCL prognosis, with the lowest degree of overfitting (**Figures 2B, C**). Decision curve analysis (DCA) was performed to evaluate the clinical utility of the LR model. The model provided a net clinical benefit across a threshold probability range of 0.05 to 1.0 for predicting 3-year mortality risk, indicating its potential clinical applicability (**Figure 2D**).

**Table 2:** Performance comparison of the DLBCL 3-year survival model built based on PET/CT depth features in the test set data.

Model	Accuracy	AUC	95% CI	Sensitivity	Specificity	PPV	NPV	F1
LR	0.865	0.950	0.907–0.993	0.875	0.863	0.600	0.967	0.712
SVM	0.825	0.892	0.826–0.957	0.708	0.853	0.531	0.926	0.607
KNN	0.849	0.786	0.672–0.901	0.417	0.951	0.667	0.874	0.513
RandomForest	0.817	0.907	0.850–0.964	0.792	0.824	0.514	0.944	0.623
XGBoost	0.897	0.830	0.744–0.917	0.500	0.990	0.923	0.894	0.649
LightGBM	0.881	0.888	0.813–0.964	0.708	0.922	0.680	0.931	0.694

PPV, Positive Predictive Value; NPV, Negative Predictive Value.

### 3.3 Performance of the clinical–PET/CT imaging fusion model

**Figure 3:**
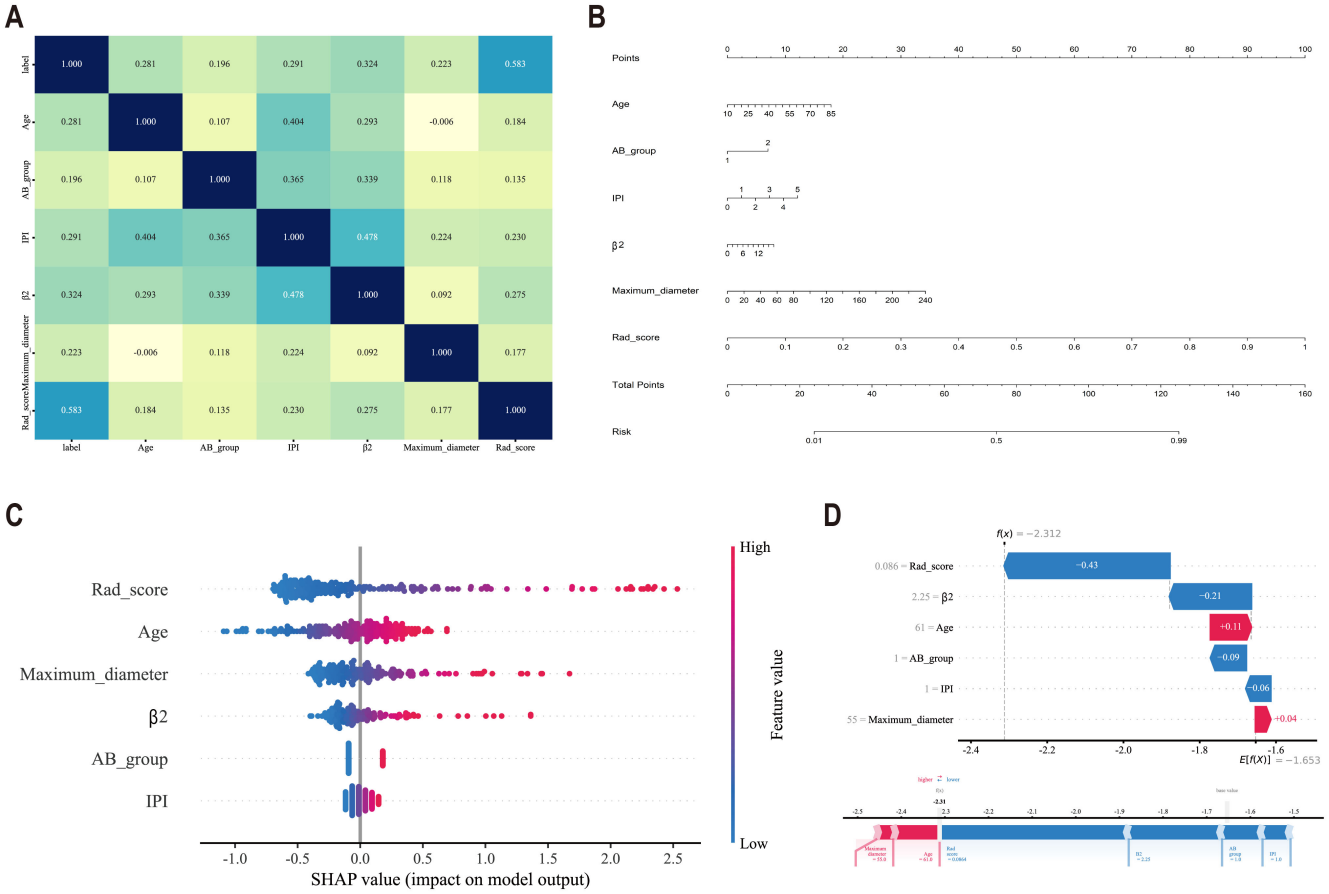
Performance of the clinical–imaging fusion model. **(A)** Correlation coefficient matrix between the radiomics score and independent clinical risk factors. **(B)** Nomogram of fusion model. **(C)** SHAP summary plot showing the overall contribution distribution and importance ranking of each clinical feature to the model prediction. **(D)** SHAP waterfall plot illustrating the contribution decomposition of each feature to the predicted value for an individual patient.

The LR algorithm, identified as the optimal classifier among the PET/CT deep learning radiomics models, was applied to predict each patient’s 3-year survival probability. This probability value was subsequently used as the radiomics score. The correlation coefficient matrix revealed no significant correlation between the radiomics score and clinically independent risk factors for 3-year survival (**Figure 3A**). A clinical–imaging fusion model was constructed by integrating the radiomics score with independent clinical features using the LR algorithm. A nomogram was developed to visually represent this model (**Figure 3B**). SHAP summary plot ranked feature importance: rad score (dominant), age, maximum tumor diameter, serum β2-microglobulin level, AB group, and IPI score. Higher feature values (red) increased predictions; lower values (blue) decreased predictions (**Figure 3C**). SHAP waterfall plot for a representative patient showed rad score (-0.43) and serum β2-microglobulin level (-0.21) were the primary negative contributors; age and maximum tumor diameter positively offset. The AB group and the IPI score had minor negative impacts. Individual attribution aligned with global analysis (**Figure 3D**). The fusion model yielded an AUC of 0.960 (95% CI: 0.895–1.000), accuracy of 0.929, sensitivity of 0.917, and specificity of 0.931, with PPV, NPV, and F1 values of 0.759, 0.979, and 0.830, respectively (**Table 3**).

**Table 3:** Comparative analysis of the discriminative performance of the clinical model, radiomic model, and fusion model.

Cohort	Model	Accuracy	AUC	95% CI	Sensitivity	Specificity	PPV	NPV	F1
Training	Radiomic model	0.867	0.889	0.805 - 0.973	0.684	0.922	0.722	0.908	0.703
	Clinical model	0.831	0.761	0.621 - 0.900	0.632	0.891	0.632	0.891	0.632
	Fusion model	0.904	0.914	0.815 - 1.000	0.789	0.937	0.789	0.937	0.789
Test	Radiomic model	0.865	0.950	0.907 - 0.993	0.875	0.863	0.600	0.967	0.712
	Clinical model	0.683	0.798	0.708 - 0.888	0.875	0.637	0.362	0.956	0.512
	Fusion model	0.929	0.960	0.895 - 1.000	0.917	0.931	0.759	0.979	0.830

**Figure 4:**
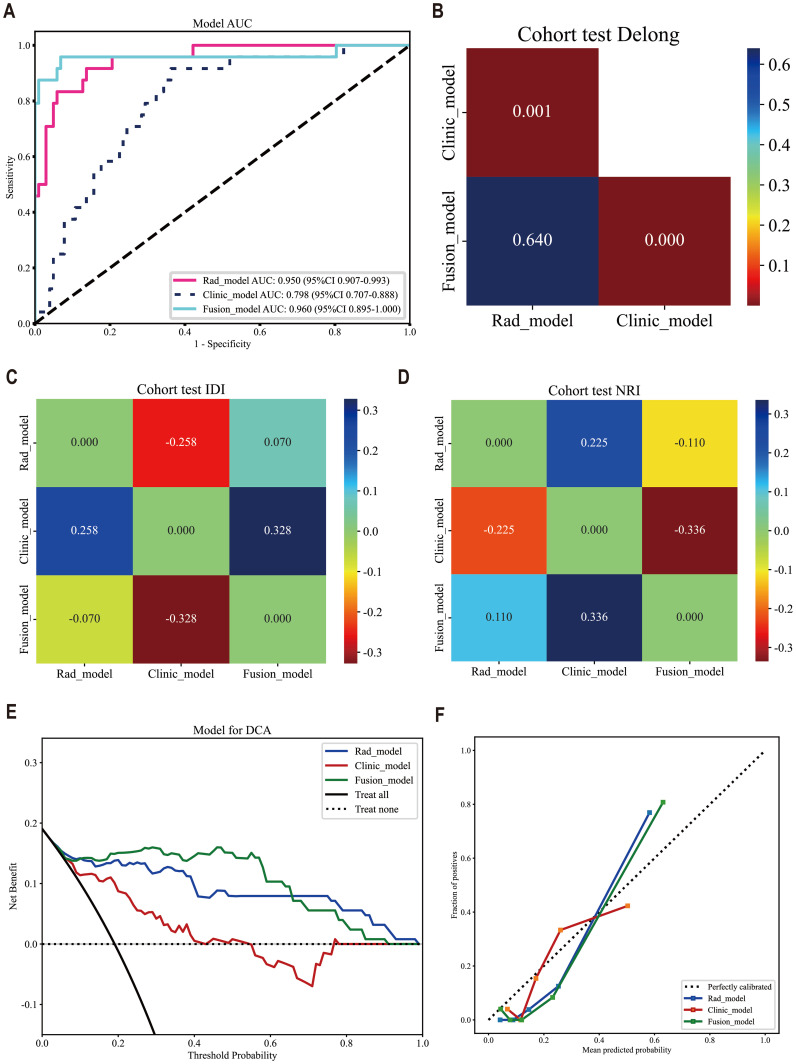
Performance evaluation of the radiomics, clinical, and fusion models in the test cohort. **(A)** ROC curves with AUC values. **(B)** Delong’s test for AUC comparison. **(C)** IDI analysis. **(D)** NRI analysis. **(E)** Decision curve analysis. **(F)** Calibration curves.

To compare the predictive efficacy of clinical and radiomic models, a clinical model was established using the same logistic regression (LR) algorithm based on five independent clinical factors: age, AB group, IPI score, serum β2-microglobulin level, and maximum tumor diameter. The predictive performance of each model is shown in **Table 3**. In the test set, the clinical model yielded an AUC of 0.798 (95% CI: 0.708-0.888), accuracy of 0.683, sensitivity of 0.875, and specificity of 0.637, with PPV, NPV, and F1 values of 0.362, 0.956, and 0.512, respectively (**Figure 4A**). Further Delong test analysis was performed to compare the AUC differences among the three models. The results demonstrated that both the fusion model and the radiomic model achieved a significantly better discriminative ability than the standalone clinical model (all P < 0.05) (**Figure 4B**). The integrated discrimination improvement (IDI) values were calculated to compare the incremental predictive performance of the three models on the test cohort. Compared with the radiomics model (Rad_model), the fusion model (Fusion_model) showed an IDI of 0.070. Compared with the clinical model, the fusion model achieved an IDI of 0.328, indicating a significant improvement in reclassification ability (**Figure 4C**). The net reclassification improvement (NRI) was calculated on the test cohort to evaluate the reclassification performance of the three models. The fusion model achieved an NRI of 0.110 over the radiomics model, demonstrating incremental predictive value. Notably, the fusion model yielded an NRI of 0.336 compared with the clinical model, representing the largest improvement in reclassification ability (**Figure 4D**). Consistently, DCA revealed that the fusion model yielded the highest net benefit across a broad range of clinically relevant threshold probabilities (0-0.90), outperforming both the radiomics and clinical models (**Figure 4E**). Calibration analysis further showed that the radiomics and fusion models exhibited good agreement between predicted probabilities and observed outcomes, with their calibration curves closely aligning with the ideal 45° line. In contrast, the clinical model showed a notable deviation from the perfectly calibrated line, particularly at higher predicted probabilities, suggesting less accurate risk estimation. Collectively, these results confirm that the fusion model provides the optimal predictive performance, reclassification ability, clinical utility, and calibration among the three models (**Figure 4F**).

### 3.4 Prognostic stratification using the fusion model

**Figure 5:**
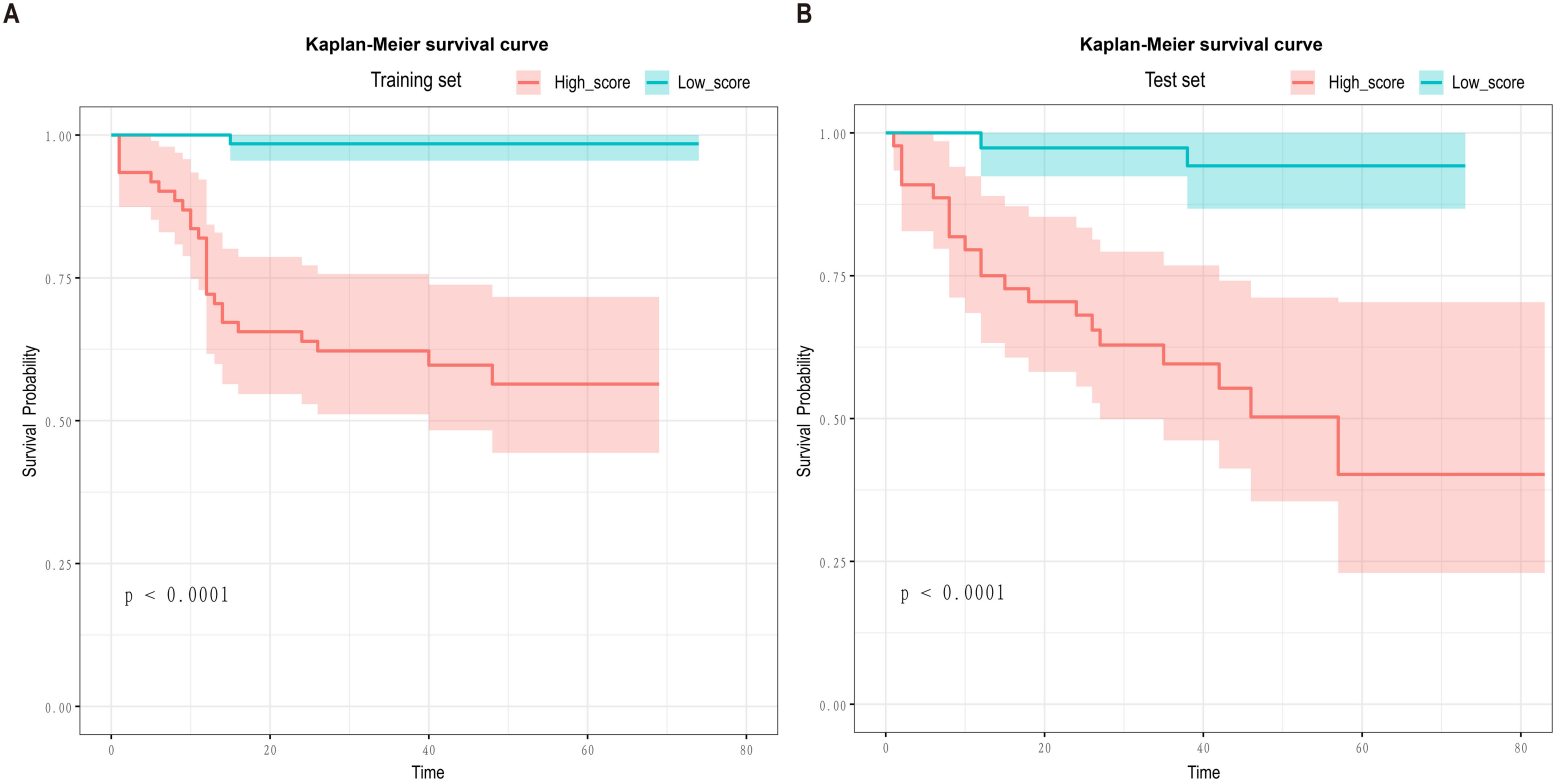
Kaplan–Meier survival curves for DLBCL patients stratified into high-score and low-score groups based on the fusion model. **(A)** Survival analysis in the training set; **(B)** Survival analysis in the test set.

Based on the median predicted probability derived from the fusion model, patients were stratified into high-score and low-score groups. Kaplan–Meier survival analysis was performed to assess differences in survival outcomes between the two groups. In both the training cohort (**Figure 5A**) and the test cohort (**Figure 5B**), the survival curve for the high-score group exhibited a gradual decline, indicating a more favorable prognosis. In contrast, the curve for the low-score group showed a steep initial drop, indicating significantly poorer survival outcomes than those in the high-score group. The log-rank test confirmed a highly significant difference in the survival distributions between the two groups (*P* < 0.0001). These findings demonstrate that risk stratification based on the clinical–imaging fusion model effectively discriminates between patient populations with distinct prognoses.

## 4 Discussion

Prognostic prediction in diffuse large B-cell lymphoma (DLBCL) has consistently been a focal point of clinical research, given the pronounced heterogeneity of the disease, which leads to substantial variability in patient outcomes (22–24). In this study, we integrated clinical characteristics with deep learning features derived from PET/CT images of 209 patients with DLBCL to construct a clinical-imaging fusion model for predicting 3-year overall survival. Our results demonstrated that this fusion model achieved an AUC of 0.960 in the test set, exhibiting excellent accuracy, specificity, and sensitivity, and significantly outperforming models based only on clinical parameters. This finding indicates that integrating multimodal data enables a more comprehensive capture of disease information, overcoming the limitations of single data sources and thereby enhancing prognostic efficacy, with substantial promise for clinical applications.

In the present study, age, AB symptoms, IPI score, serum β2-microglobulin level, and maximum tumor diameter were confirmed as independent prognostic factors for DLBCL, consistent with the majority of previous reports (25–27). Age is a pivotal factor influencing prognosis (28). Thus, age not only directly impacts survival outcomes but may also indirectly influence prognosis through its association with the expression of biomarkers. In our analysis, age emerged as the most significant predictor of 3-year survival, underscoring its critical role in the prognostic assessment. The presence of B symptoms (fever, night sweats, and weight loss) and manifestations of systemic disease are correlated with poorer OS and PFS (29). In our cohort, the proportion of patients presenting with B symptoms was significantly higher in the non-survival group than in the survival group (51.2% vs. 28.3%, P = 0.0177), further supporting B symptoms as an indicator of poor prognosis in DLBCL. The International Prognostic Index (IPI) remains a cornerstone tool for DLBCL prognostication and is widely applied in clinical practice for risk stratification and therapeutic decision-making. The IPI score is directly correlated with survival outcomes. A large-scale study of Chinese DLBCL patients demonstrated that individuals with IPI scores of 4–5 had significantly inferior survival rates compared to those with lower scores, with high IPI scores predicting worse OS and PFS (22). Our study further validates the IPI as an independent prognostic determinant. Serum β2-microglobulin levels are associated with tumor burden and microenvironmental inflammation. An investigation into HIV-related DLBCL revealed that patients with serum β2-microglobulin levels ≥5 mg/L had significantly poorer OS than those with lower levels, suggesting its utility as an independent prognostic factor (30). A maximum tumor diameter exceeding 7.5 cm (bulky disease) suggests high proliferative activity and a hypoxic tumor microenvironment, both of which are closely linked to adverse outcomes (31). These factors may synergistically contribute to tumor heterogeneity, immune evasion, and metabolic dysregulation, collectively shaping the prognosis of patients. Nevertheless, traditional prognostic systems still fall short of the clinical requirement for the precise identification of high-risk DLBCL patients, necessitating the integration of novel technologies to address the disease’s molecular heterogeneity and complexity.

In recent years, artificial intelligence (AI), particularly deep learning, has profoundly transformed medical image analysis, particularly in the precision diagnosis and management of neoplastic diseases (16, 32). In this study, we developed a multimodal PET/CT-based model for the prognostic prediction of 209 patients with DLBCL. Among the radiomic models constructed using deep features fused from PET and CT images, the logistic regression (LR) model demonstrated optimal predictive performance for DLBCL prognosis, achieving high accuracy (0.865), sensitivity (0.875), and specificity (0.863). This indicates the robust feasibility of our model for assessing the 3-year survival risk in patients with DLBCL. Deep features extracted from multimodal imaging, such as PET/CT, using deep learning have shown immense potential to surpass traditional imaging features, providing more powerful tools for cancer diagnosis and prognosis (33). Numerous studies have substantiated the superiority of deep features in oncological diagnoses. For instance, research has shown that features extracted from PET/CT images using deep learning models can enhance the accuracy of recurrence-free survival analysis in patients with head and neck cancer, outperforming conventional manual segmentation methods (34). In prostate cancer, multimodal deep learning models integrating PET/CT and multiparametric MRI have exhibited excellent predictive capabilities for adverse pathological features, significantly outperforming single-modality models (35). Furthermore, in lung cancer and lymphoma, combining PET and CT information using 3D-CNNs enables high-precision anatomical localization and classification, substantially boosting diagnostic performance, with deep features achieving AUC values of 0.97-0.99 (36). More critically, regarding the core clinical issue of prognosis prediction, deep features have demonstrated enhanced predictive power. A study employing deep learning models combined with quantitative magnetic resonance imaging features successfully predicted biochemical recurrence following radical prostatectomy for prostate cancer, achieving a C-index of 0.802, which was significantly superior to that of traditional clinical models (37). Deep learning has also excelled in predicting the pathological complete response in breast cancer. Through deep learning analysis of histopathological tissue sections, researchers have developed a novel biomarker for predicting pathological complete response that outperforms traditional biomarkers (38). Moreover, deep learning techniques have been preliminarily explored for DLBCL. Previous research investigating the diagnostic and prognostic value of baseline FDG PET/CT-derived skeletal textural features (TFs) in DLBCL has revealed their significant utility in DLBCL. One study demonstrated that skeletal TFs exhibited superior sensitivity (81.8%) and specificity (81.7%) in identifying bone marrow involvement (BMI), particularly in patients with negative bone marrow biopsy (BMB) and negative PET visual analysis, suggesting their potential value in detecting BMI overlooked by conventional methods (39). Chang et al., in a study of 122 DLBCL patients, used a deep neural network (DNN) to predict PFS and OS, achieving accuracies of 71% and 76%, respectively (40). While PET/CT is the gold standard for staging and response assessment in DLBCL, and traditional metabolic parameters such as SUVmax, TLG, and MTV quantitatively assess tumor activity and treatment response (41), information from single-modality PET or CT images is limited and not as reliable. They fail to capture the spatial heterogeneity within tumors, and lesion delineation (especially for diffuse disease) is subject to inter-observer variability, hindering further improvements in the prognostic accuracy. Therefore, researchers have increasingly focused on integrating PET and CT images to create multimodal datasets, with multimodal radiomic models demonstrating their superiority across various cancer types (42, 43).

The application of AI has further augmented the predictive capacity derived from combining imaging features with clinical data, demonstrating significant advantages in the survival prediction of malignancies. Hong et al. confirmed that multimodal models integrating radiomic and clinical features demonstrated superior predictive performance for postoperative survival in patients with colorectal cancer (44). Other investigators have explored AI-based models integrating multimodal data, including CT and ultrasound images along with patient clinical data, to successfully predict survival in patients with solid tumors. These models demonstrated significant advantages in distinguishing high-risk patients from low-risk patients, indicating that combining imaging features with clinical data enables effective patient risk stratification (45). In the current study, we constructed and validated a clinical-imaging fusion model that integrates clinical features with PET/CT-derived deep features to predict 3-year survival in patients with DLBCL. Our experimental results revealed that this fusion model achieved AUC values of 0.914 and 0.960 in the training and test sets, respectively, demonstrating a significantly superior predictive performance compared to single-modality models. This finding suggests that the effective integration of multimodal information can provide a more comprehensive reflection of tumor biology, thereby enhancing prognostic accuracy. We attribute this performance improvement to the synergistic effect of deep learning’s capacity to automatically extract high-dimensional latent features from images, complemented by clinical indicators that provide systemic information not readily captured by imaging alone. A recent study by Dondolin et al. demonstrated that combining PET radiomics with liquid biopsy (ctDNA) improves outcome prediction in DLBCL (46). Given that our current model already integrates PET/CT deep features with clinical variables, we believe that future research should extend this framework to incorporate ctDNA genomics and pathomics. Such multi-omics, multi-dimensional fusion would capture complementary biological information, thereby enhancing model robustness, generalizability, and clinical utility.

Despite the methodological strengths and promising performance of our model, several limitations warrant consideration. First, this was a single-center retrospective study, which may have introduced selection bias and confounding factors into the results. The model’s validity was established solely through internal validation; therefore, external validation across multicenter datasets is imperative to assess its generalizability. Second, the relatively modest sample size may affect the model’s stability and broader applicability, and may introduce sampling variability. Future studies with larger prospective cohorts are needed to validate the model’s stability and generalizability. Third, the current feature extraction process relies on manual delineation, which limits workflow automation and standardization. Fourth, due to the limited overall sample size, we were unable to perform subgroup analyses based on different genetic mutations or clinical treatment response subtypes to further validate the performance of our PET/CT-based deep learning model. Additionally, this study lacked comparative analysis with well-recognized PET metabolic biomarkers, including TMTV and TLG. Although we contoured the largest metabolically active lesion to extract deep learning features, whole-body delineation of all PET-positive lesions for TMTV and TLG quantification. Accordingly, a direct head-to-head comparison between our predictive model and conventional metabolic biomarkers was unavailable. Future investigations with full-body PET segmentation and TMTV/TLG calculation are required to further verify the incremental value of our model.

## 5 Conclusions

The radiomic model constructed using deep features extracted from PET/CT images demonstrates feasibility for predicting 3-year survival risk in DLBCL patients. Fusing this model with pertinent clinical prognostic features further enhanced its accuracy, specificity, and sensitivity. This integrated fusion model may serve as a reliable tool for assessing the prognosis of patients with DLBCL, thereby aiding clinicians in formulating individualized treatment strategies.

## Data Availability

The data supporting the findings of this study are not publicly available due to restrictions imposed by the Institutional Review Board of the First Affiliated Hospital of Guangxi Medical University to protect participant privacy. De-identified data may be made available to qualified researchers upon reasonable request, subject to approval from the Institutional Review Board and execution of a signed data access agreement. Requests to access these datasets should be directed to ZP, Drpzg001@163.com.
