# Supplementary material for: A deep learning-based prognostic model for diffuse large B-cell lymphoma incorporating PET/CT imaging features
Source: Front Oncol. 2026 Jun 16;16:1849942. doi: 10.3389/fonc.2026.1849942 (PMC13314506; doi:10.3389/fonc.2026.1849942)
Supplement: Supplementary file 1 [file Table1.docx]

Supplemental Table 1 Comparison of baseline data on 3-year survival of DLBCL

| **Characteristic** | ****Survival Group****  **(N=166)** | ****Non-survival Group****  **(N=43)** | ****Total****  **(N=209)** | ***P***value**** |
| --- | --- | --- | --- | --- |
| **Gender , n (%)** |  |  |  | 0.12 |
| Male | 75 (45.2%) | 27 (62.8%) | 102 (48.8%) |  |
| Female | 91 (54.8%) | 16 (37.2%) | 107 (51.2%) |  |
| **Age, years** | 52.6 (14.0) | 61.9 (12.5) | 54.5 (14.2) | <0.001 |
| **GCB Subtype, n (%)** |  |  |  | 0.877 |
| non-GCB | 121 (72.9%) | 33(76.7%) | 154 (73.7%) |  |
| GCB | 45 (27.1%) | 10 (23.3%) | 55(26.3%) |  |
| **Ann Arbor Stage, n (%)** |  |  |  | 0.277 |
| I | 15 (9.0%) | 3 (7.0%) | 18 (8.6%) |  |
| II | 43(25.9%) | 4 (9.3%) | 47 (22.5%) |  |
| III | 17 (10.2%) | 3 (7.0%) | 20 (9.6%) |  |
| IV | 91 (54.8%) | 33 (76.7%) | 124 (59.3%) |  |
| AB_group**, n (%)** |  |  |  | 0.0177 |
| Group A | 119 (71.7%) | 21.0 (48.8%) | 140 (67.0%) |  |
| Group B | 47 (28.3%) | 22 (51.2%) | 69 (33.0%) |  |
| **International Prognostic Index (IPI)** | 1.89 (1.40) | 2.98 (1.42) | 2.11 (1.47) | <0.001 |
| **Hemoglobin(HGB), g/dL** | 114 (24.0) | 106 (21.2) | 112 (23.6) | 0.159 |
| **Neutrophil (NEU), ×10⁹/L** | 4.26 (2.26) | 5.30 (3.79) | 4.47 (2.67) | 0.0743 |
| **Lymphocyte (LYM), ×10⁹/L** | 1.72 (3.91) | 1.50 (1.38) | 1.67 (3.54) | 0.939 |
| **Monocyte(MON), ×10⁹/L** | 1.28 (6.91) | 0.995 (0.706) | 1.22 (6.16) | 0.965 |
| **Lactate Dehydrogenase (LDH), U/L** | 342 (398) | 541 (696) | 383 (479) | 0.052 |
| **β2-Microglobulin (β2-MG), mg/L** | 3.75 (2.28) | 5.56 (3.17) | 4.12 (2.59) | <0.001 |
| **Maximum Tumor Diameter, mm** | 49.4 (39.6) | 71.3 (47.0) | 53.9 (42.0) | 0.0093 |
| SUVmax | 17.1 (9.92) | 19.5 (9.46) | 17.6 (9.85) | 0.385 |
