# Supplementary material for: A deep learning-based prognostic model for diffuse large B-cell lymphoma incorporating PET/CT imaging features
Source: Front Oncol. 2026 Jun 16;16:1849942. doi: 10.3389/fonc.2026.1849942 (PMC13314506; doi:10.3389/fonc.2026.1849942)
Supplement: Supplementary file 2 [file Table2.docx]

Supplemental Table 2 Comparison of baseline data of model construction training set and test set

|  | **Training set**  **(N=146)** | **Test set**  **(N=63)** | ***P***value**** |
| --- | --- | --- | --- |
| **Gender , n (%)** |  |  | 0.710 |
| Male | 74 (50.7%) | 28 (44.4%) |  |
| Female | 72 (49.3%) | 35 (55.6%) | 0.472 |
| **Age, years** | 55.3 (14.1) | 52.7 (14.2) |  |
| **GCB Subtype, n (%)** |  |  | 0.677 |
| non-GCB | 105 (71.9%) | 49 (77.8%) |  |
| GCB | 41 (28.1%) | 14 (22.2%) |  |
| **Ann Arbor Stage, n (%)** |  |  | 0.987 |
| I | 14 (9.6%) | 4 (6.3%) |  |
| II | 32 (21.9%) | 15 (23.8%) |  |
| III | 15 (10.3%) | 5 (7.9%) |  |
| IV | 85 (58.2%) | 39 (61.9%) |  |
| AB_group**, n (%)** |  |  | 0.404 |
| Group A | 102 (69.9%) | 38 (60.3%) |  |
| Group B | 44 (30.1%) | 25 (39.7%) |  |
| **International Prognostic Index (IPI)** | 2.14 (1.48) | 2.03 (1.47) | 0.880 |
| **Hemoglobin(HGB), g/dL** | 115 (22.4) | 106 (25.5) | 0.059 |
| **Neutrophil (NEU), ×10⁹/L** | 4.40 (2.24) | 4.62 (3.49) | 0.862 |
| **Lymphocyte (LYM), ×10⁹/L** | 1.49 (1.08) | 2.10 (6.24) | 0.518 |
| **Monocyte(MON), ×10⁹/L** | 1.44 (7.36) | 0.704 (0.586) | 0.731 |
| **Lactate Dehydrogenase (LDH), U/L** | 390 (528) | 368 (343) | 0.953 |
| **β2-Microglobulin (β2-MG), mg/L** | 4.03 (2.52) | 4.34 (2.75) | 0.724 |
| **Maximum Tumor Diameter, mm** | 56.5 (44.6) | 47.8 (35.1) | 0.387 |
| SUVmax | 17.8 (9.56) | 17.2 (10.5) | 0.921 |
| **3-Year Survival** |  |  | 1 |
| NO | 116 (79.5%) | 50 (79.4%) |  |
| Yes | 30 (20.5%) | 13 (20.6%) |  |
