# Supplementary material for: A deep learning-based prognostic model for diffuse large B-cell lymphoma incorporating PET/CT imaging features
Source: Front Oncol. 2026 Jun 16;16:1849942. doi: 10.3389/fonc.2026.1849942 (PMC13314506; doi:10.3389/fonc.2026.1849942)
Supplement: Supplementary file 3 [file Table3.docx]

Supplemental Table 3 Performance comparison of DLBCL 3-year survival model built based on PET/CT depth features in training set data

| **Model** | ****Accuracy**** | **AUC** | **95% CI** | ****Sensitivity**** | ****Specificity**** | **PPV** | **NPV** | **F1** |
| --- | --- | --- | --- | --- | --- | --- | --- | --- |
| LR | 0.867 | 0.889 | 0.805 - 0.973 | 0.684 | 0.922 | 0.722 | 0.908 | 0.703 |
| SVM | 0.880 | 0.904 | 0.811 - 0.997 | 0.789 | 0.906 | 0.714 | 0.935 | 0.750 |
| KNN | 0.795 | 0.876 | 0.801 - 0.951 | 0.684 | 0.828 | 0.542 | 0.898 | 0.605 |
| RandomForest | 0.976 | 1.000 | 1.000 - 1.000 | 0.895 | 1.000 | 1.000 | 0.970 | 0.944 |
| XGBoost | 0.964 | 0.998 | 0.994 - 1.000 | 0.947 | 0.969 | 0.900 | 0.984 | 0.923 |
| LightGBM | 0.831 | 0.914 | 0.850 - 0.977 | 0.684 | 0.875 | 0.619 | 0.903 | 0.650 |

PPV:**Positive Predictive Value.** NPV:**Negative Predictive Value**
